# Letter to the Editor for the article ‘A nomogram incorporating treatment data for predicting overall survival in gastroenteropancreatic neuroendocrine tumors: a population-based cohort study’

**DOI:** 10.1097/JS9.0000000000001648

**Published:** 2024-05-20

**Authors:** Pengcheng Zhang, Lexin Wang, Ke Xu, Gang Tian

**Affiliations:** a The First Affiliated Hospital of Zhejiang Chinese Medical University (Zhejiang Provincial Hospital of Chinese Medicine), Hangzhou; b General Hospital of Ningxia Medical University; c Ningxia Medical University, Yinchuan, Ningxia; d Department of Oncology, Chongqing General Hospital, Chongqing University, Chongqing; e Department of Clinical Laboratory Medicine, The Affiliated Hospital of Southwest Medical University, Luzhou, People’s Republic of China

*Dear Editor,*

We are grateful for the opportunity to discuss the manuscript, ‘A nomogram incorporating treatment data for predicting overall survival in gastroenteropancreatic neuroendocrine tumors: a population-based cohort study,’ in the *International Journal of Surgery*. This study innovatively constructs a nomogram that integrates treatment data to prognosticate overall survival (OS) in patients with gastroenteropancreatic neuroendocrine tumors (GEP-NETs). Leveraging the Surveillance, Epidemiology, and End Results (SEER) database, the study employs multivariate Cox regression analysis to identify prognostic factors, demonstrating the superior predictive capacity of our nomogram over the existing 7th edition of the American Joint Committee on Cancer (AJCC) staging system. This tool promises to enhance clinical decision-making, tailor treatments individually, and improve patient quality of life.

Despite its novelty, the study acknowledges limitations. The nomogram does not incorporate all prognostic factors, such as significant biomarkers like Ki-67 and TP53^1^ or predictive markers for immunotherapy, which guide the treatment of GEP-NETs. Future iterations should enhance the model by integrating additional databases for a more nuanced classification.

Clinical-pathological markers such as recurrence-free survival, microvascular invasion, and tumor infiltration depth^2,3^, which may influence predictive accuracy, were not discussed. The association between radiation therapy and poor prognosis should be interpreted with caution due to the limited sample size of patients receiving radiation; we recommend validation through prospective studies and independent patient cohorts to bolster evidence.

Artificial intelligence techniques, including radiomics, pathomics, and applications of transcriptomics and single-cell sequences, could significantly supplement and validate the model. Future upgrades may benefit from machine learning approaches like Support Vector Machine (SVM), Multilayer Perceptron (MLP), and Decision Tree (DT) for data selection, thus refining the nomogram’s predictive precision.

In conclusion, despite these limitations, our study provides a novel and valuable tool for prognostic assessment in GEP-NET patients and lays the groundwork for future exploration in personalized medicine.

## Ethical approval

Not applicable.

## Consent

All the authors consent for the publication of the article.

## Sources of funding

This study was supported by grants from the Luzhou Science and Technology Department Applied Basic Research Program (No: 2022-JYJ-145), the Sichuan Province Science and Technology Department of foreign (border) high-end talent introduction project (No: 2023JDGD0037), Sichuan Provincial Medical Association (No: Q22027), and Zhejiang Provincial Traditional Chinese Medicine Science and Technology Program (No: 2023ZL467).

## Author contribution

P.Z., L.W., K.X., and G.T.: conceptualization, methodology, validation, formal analysis, investigation, resources, data curation, writing – original draft, writing – review and editing, supervision, and project administration.

## Conflicts of interest disclosure

The authors declare that they have no conflicts of interest.

## Research registration unique identifying number (UIN)

Not applicable.

## Guarantor

Pengcheng Zhang, Lexin Wang, Ke Xu, and Gang Tian.

## Data availability statement

Data sharing is not applicable to this article as no new data were created or analyzed in this study.

## Provenance and peer review

Not applicable.
